# A phase 1 randomized study compare the pharmacokinetics, safety and immunogenicity of HLX04 to reference bevacizumab sourced from the United States, the European Union, and China in healthy Chinese male volunteers

**DOI:** 10.1007/s00280-021-04297-z

**Published:** 2021-06-04

**Authors:** Xiaoxue Zhu, Hongjie Qian, Jixuan Sun, Min Wu, Chen Yu, Yanhua Ding, Xiaodi Zhang, Katherine Chai, Xiaojiao Li

**Affiliations:** 1 The First Hospital of Jilin University, No. 1 Xinmin Street, Chaoyang District, Changchun, Jilin China; 2 Central Laboratory, Shanghai Xuhui Central Hospital, Shanghai, China; 3 Shanghai Engineering Research Center of Phase I, Clinical Research & Quality Consistency Evaluation for Drugs, Shanghai, China; 4 Shanghai Henlius Biotech, Inc., Shanghai, China

**Keywords:** Bevacizumab, Biosimilar, Pharmacokinetics, Bioequivalence

## Abstract

**Purpose:**

To compare the pharmacokinetic profiles, safety and immunogenicity of proposed bevacizumab biosimilar HLX04 with reference bevacizumab in healthy Chinese males.

**Methods:**

In this double-blind Phase 1 study, healthy volunteers (*N* = 208) were randomized 1:1:1:1 to a single 3 mg/kg intravenous infusion of HLX04 or reference bevacizumab sourced from the United States (bevacizumab-US), the European Union (bevacizumab-EU) or China (bevacizumab-CN). Co-primary endpoints were area under the serum concentration–time profile (AUC) from time zero extrapolated to infinity (AUC_0–inf_) and from zero to last quantifiable concentration (AUC_last_). Secondary endpoint was the maximum serum drug concentration (C_max_). Study participants were monitored for treatment-emergent adverse events (TEAEs) and samples were collected for anti-drug antibody (ADA) testing throughout the study.

**Results:**

Pharmacokinetic parameters were similar across groups. The respective geometric least-squares mean ratios (GLSMR) of AUC_0–inf_, AUC_last_ and C_max_ were: 95.7%, 96.0% and 101.8% for HLX04 versus bevacizumab-US; 94.3%, 94.6% and 100.5% for HLX04 versus bevacizumab-EU; and 90.0%, 90.4% and 98.2% for HLX04 versus bevacizumab-CN. For all test-to-reference comparisons, two-sided 90% confidence intervals of GLSMR for AUC_0–inf_, AUC_last_ and C_max_ fell in the pre-specified bioequivalence range (80–125%). There were no notable differences in the frequency, nature and/or grade of TEAEs. No deaths were reported and no ADAs were detected during the study.

**Conclusion:**

HLX04 had similar safety and pharmacokinetic profiles to reference bevacizumab in healthy Chinese males, supporting the confirmatory Phase 3 study investigating the efficacy and safety equivalence between HLX04 and bevacizumab in patients with metastatic colorectal cancer (NCT03511963).

**Clinical trial registration:**

The study was registered with Clinicaltrials.gov, NCT03483649.

**Supplementary Information:**

The online version contains supplementary material available at 10.1007/s00280-021-04297-z.

## Introduction

Bevacizumab (Avastin^®^; Genentech, Inc., San Francisco, USA; Roche Pharma AG, Grenzach-Wyhlen, Germany) is a recombinant, humanized, monoclonal immunoglobulin G1 (IgG1) antibody that binds to human vascular endothelial growth factor (VEGF) [1, 2]. The regular function of VEGF is to promote angiogenesis and vascularization, a process which is hijacked during tumor growth and metastasis [1, 2]. VEGF mRNA is overexpressed in the majority of human tumors [1, 2].

Bevacizumab has been approved since 2004 for the treatment of several solid tumors, including metastatic colorectal cancer (mCRC), ovarian cancer, cervical cancer, non-squamous cell non-small cell lung cancer (NSCLC), renal cell carcinoma and glioblastoma [3–5]. In China, bevacizumab was approved since 2010 for the treatment of mCRC and since 2015 for the treatment of NSCLC [6]. According to Avastin^®^ FDA label, the mean [% coefficient variation (CV)] central volume of distribution of bevacizumab is 2.9 (22%) L [7]. The mean (CV%) clearance and estimated half-life of bevacizumab are 0.23 (33%) L/day and 20 days (11–50 days), respectively [7].

Biosimilars are defined as biologic medical products that are highly similar to an approved reference product, notwithstanding minor clinically insignificant differences [8, 9]. The clinical needs for affordable bevacizumab treatment have resulted in the development of a number of biosimilar molecules [10–21]. As of August 2020, two bevacizumab biosimilars (Zirabev^®^ and Mvasi^®^) have been approved by the United States Food and Drug Administration (FDA) [22, 23] and the European Medicines Agency (EMA) [24, 25], and two (Byvasda^®^ and Ankada^®^) by the Chinese National Medical Products Administration (NMPA) [26, 27]. The bevacizumab biosimilars will provide patients with high-quality alternatives and potentially increase the patient accessibility.

The recombinant, humanized monoclonal antibody HLX04 was developed as a bevacizumab biosimilar. Preclinical studies have confirmed a high degree of similarity between HLX04 and reference bevacizumab in terms of structure, physicochemical characteristics and in vitro biological activity. Furthermore, in vivo testing has demonstrated that HLX04 has very similar pharmacokinetic and toxicokinetic characteristics to bevacizumab in cynomolgus monkeys at doses of 2–50 mg/kg [28].

In accordance with the standard stepwise approach of biosimilar development [8, 9, 29], here we report the clinical pharmacokinetic, safety and immunogenicity results collected from healthy Chinese males. The primary objective of this study was to determine pharmacokinetic bioequivalence of HLX04 with reference bevacizumab sourced from the United States (bevacizumab-US), the European Union (bevacizumab-EU) or China (bevacizumab-CN). Secondary objectives were to evaluate the safety, tolerability and immunogenicity of HLX04.

## Methods

### Study design and ethics

**Fig. 1:**
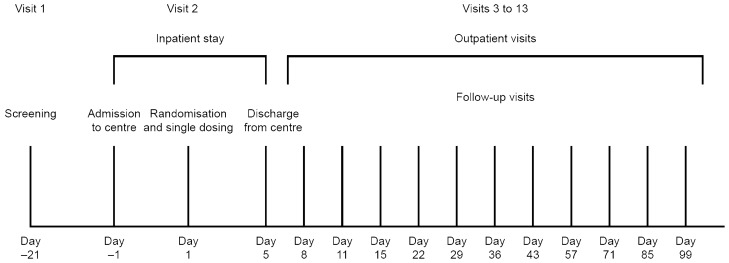
Study design

This Phase 1, randomized, double-blind, single-dose, four-arm, parallel-controlled study was conducted in healthy Chinese male volunteers (Fig. 1). Following a 21-day screening period, eligible individuals were admitted to the clinical trial units at Shanghai Xuhui Central Hospital (*n* = 18) and the First Hospital of Jilin University (*n* = 190). On Day 1, participants were randomized 1:1:1:1 via an interactive web-response system to receive a single 3 mg/kg intravenous infusion of HLX04, bevacizumab-US (Genentech, Inc.), bevacizumab-EU (Roche Pharma AG) or bevacizumab-CN [Roche Pharma (Schweiz) Ltd.]. Study participants and personnel were blinded to the assigned treatment. Study drugs were administered over a 90-min infusion period. All participants were fasted overnight for at least 8 h prior to dosing and for 4 h after the start of the infusion. Water was permitted ad libitum. Participants were required to refrain from strenuous exercise for 72 h prior to screening, until 60 days after administration. For the first eight participants, a maximum of one individual per day received study treatment and the safety profiles of preceding participants were reviewed by the principal investigator prior to each administration. After the study treatments received by the first eight individuals were deemed safe, the enrollment and treatment of all remaining participants was permitted. Study participants were required to remain in the clinical trial unit until Day 5 for safety evaluation, after which they were discharged. Follow-up visits for safety, pharmacokinetics and immunogenicity assessments were performed.

This study was conducted in accordance with the Declaration of Helsinki, NMPA regulations and the International Conference on Harmonisation E6 Guideline for Good Clinical Practice. An independent ethics committee at each study site [Shanghai Xuhui Central Hospital (approval number: 2016-38) and the First Hospital of Jilin University (approval number: 160831-168)] approved the study protocol and subsequent amendments.

### Participants’ eligibility criteria

Healthy males aged 18–50 years, with body mass index of 19–26 kg/m^2^ and body weight of 50–80 kg were eligible for this study. All participants were non-smokers or had a smoking history of < 5 cigarettes/day and had not used tobacco- or nicotine-containing products for ≥ 3 months prior to screening. Individuals were excluded if they had: current, or a history of, any clinically significant medical condition or malignancy; a history of exposure to any investigational monoclonal antibody in the 12 months prior to the study, or any prior exposure to a monoclonal antibody or protein targeting VEGF or the VEGF receptor; a history of drug or alcohol abuse, or a positive urine drug screen or alcohol breath test during screening; surgery in the past 8 weeks or planned during the study; intake of a non-steroidal anti-inflammatory drug (including aspirin) within 14 days; intake of any prescribed or over-the-counter drug, herbal drug or dietary supplement within 28 days, or use of any investigational drug in the context of a clinical study within 3 months prior to receiving study treatment; or any significant blood loss, or blood donation or transfusion in the 3 months prior to screening. Full inclusion and exclusion criteria could be found in the Supplementary table 1.

### Study endpoints

The primary pharmacokinetic endpoints were area under the serum concentration–time profile (AUC) from time zero extrapolated to infinite time (AUC_0–inf_) and from zero to the last time point with quantifiable concentration (AUC_last_). AUC_0-inf_ and AUC_last_ were calculated using the linear-up log-down method. The secondary pharmacokinetic endpoint was maximum serum drug concentration (C_max_). Other key pharmacokinetic endpoints were time to reach C_max_ (t_max_), terminal half-life (t_1/2_), total body clearance (CL) and volume of distribution at steady state (V_d_). Safety outcomes were treatment-emergent adverse events (TEAEs) including serious TEAEs and infusion-site reactions, clinical laboratory parameters, vital signs, 12-lead electrocardiogram (ECG) and physical examination. The incidence of anti-drug antibodies (ADAs) was the sole immunogenicity endpoint.

### Pharmacokinetic evaluations

Blood samples for pharmacokinetic analysis were collected on Day 1, 1 h before the start of infusion, 1.5 h after the start of infusion, at 1, 2, 4 and 8 h after the end of infusion; on Days 2, 3, and 5 and at every follow-up visit thereafter. Serum concentrations of HLX04 and bevacizumab were assessed via an enzyme-linked immunosorbent assay (ELISA) and analyzed by bioanalytical laboratory WuXi AppTec (Shanghai) Co., Ltd. The standard curve was defined between 28.21 and 1504 ng/mL. During sample analysis, the precision (expressed as the CV% of quality control samples) was 6.6% to 11.0% and the accuracy [expressed as the percentage relative error (% RE) of quality control samples] was − 8.0 to 2.2%.

### Immunogenicity evaluations

Samples were collected for ADA testing during the screening period and on Days 15, 43, 71 and 99. Individuals with positive ADA samples were followed up for 12 months after the end of study visit, or until two consecutive samples were tested negative. The concentration of circulating ADAs was assessed via electrochemiluminescent (ECL) assay at WuXi AppTec (Shanghai) Co., Ltd. The sensitivity of this ECL assay is 16 ng/mL. During sample analysis, the precision was below 16.2%.

### Safety evaluations

Study participants were monitored for TEAEs throughout the study. Physical examinations were carried out at screening and on Days 2, 5, 8, 11, and 15. Then, if clinically indicated and at end of study, vital signs were taken at screening and at multiple time points during Day 1, twice daily on Days 2 and 3, then on Days 5, 8, 11, 15, 43 and 99. A 12-lead ECG was undertaken at rest during screening and on Days 1, 2, 5, 11 and 99. Clinical laboratory tests (hematology, coagulation, chemistry and urinalysis) were performed during screening and on Days 3, 11, 29, 43, 57, 71 and 99.

### Sample size and statistical methods

Estimation of sample size was based on previous studies [12, 30] and allowed for ~ 10% of study participants with non-evaluable pharmacokinetic data, and inter-subject variability of 25%. A sample size of 208 evaluable individuals (47 per treatment group) was estimated using Bonferroni adjustment to provide 80% joint power (96% power for each individual comparison) to demonstrate pharmacokinetic bioequivalence within the pre-specified 80‒125% limit [31, 32], if the ratio of true means was 1.05 (at the *α* = 0.05 level for two one-sided *t* tests).

Pharmacokinetic parameters were calculated using non-compartmental methods for all study participants who completed the study with an evaluable HLX04 or bevacizumab serum concentration–time profile for both primary endpoints, and who did not experience any major protocol deviations or important events with the potential to affect pharmacokinetic assessment (pharmacokinetic analysis population). Non-compartmental computation of pharmacokinetic parameters was performed using Phoenix^®^ WinNonlin^®^ version 6.4 (Pharsight Corporation, a Certara Company, Princeton, NJ, USA).

The point estimate and two-sided 90% confidence interval (CI) for the geometric least-squares mean ratios (GLSMR) for C_max_, AUC_0–inf_ and AUC_last_ were estimated using an analysis of variance (ANOVA) model with treatment as a fixed effect. Five comparisons were performed: HLX04 versus bevacizumab-US, HLX04 versus bevacizumab-EU, HLX04 versus bevacizumab-CN, bevacizumab-US versus bevacizumab-EU, and bevacizumab-CN versus bevacizumab-EU. Pharmacokinetic biosimilarity was concluded if the two-sided 90% CI of the test-to-reference GLSMR of AUC_0–inf_, AUC_last_ and C_max_ fell in the pre-specified 80‒125% bioequivalence limit. Pharmacokinetic data were log-transformed prior to statistical analysis.

TEAEs, defined as AEs that first occurred or worsened in severity after study drug administration, and other safety outcomes, were recorded in all treated participants (safety analysis population). TEAEs were coded and grouped using Medical Dictionary for Regulatory Activities, version 19.1, and summarized by severity and relationship to treatment. AEs were graded according to National Cancer Institute Common Terminology Criteria for Adverse Events version 4.03.

All statistical outputs were generated using SAS^®^ version 9.4 (SAS Institute Inc., Cary, NC, USA).

## Results

### Disposition and characteristics of study participants

This trial was conducted between April 12, 2017 and November 09, 2017. A total of 208 individuals were enrolled and randomized to study treatment (*n* = 52 per treatment group: HLX04, bevacizumab-US, bevacizumab-EU and bevacizumab-CN). Baseline characteristics and demographics were well balanced among groups (Table 1). Ages of participants ranged from 19 to 50 years, weight ranged from 50.2 to 79.8 kg, and height ranged from 154 to 182 cm in this study.

**Table 1:** Baseline characteristics and demographics of the study participants (randomized population)

Description	HLX04 (*n* = 52)	BV-US (*n* = 52)	BV-EU (*n* = 52)	BV-CN (*n* = 52)	Total (N = 208)
Mean age, years (SD)	37.1 (8.9)	39.4 (7.9)	37.3 (8.7)	38.7 (8.9)	38.1 (8.6)
Median age, years (range)	36.0 (22–50)	41.5 (22–50)	39.0 (19–49)	42.0 (22–49)	39.5 (19–50)
Race, *n* (%)					
Han Chinese	50 (96.2)	51 (98.1)	45 (86.5)	50 (96.2)	196 (94.2)
Non-Han Chinese	2 (3.8)	1 (1.9)	7 (13.5)	2 (3.8)	12 (5.8)
Mean height, cm (SD)	169.0 (4.7)	167.4 (6.5)	167.7 (5.8)	167.3 (5.8)	167.8 (5.7)
Median height, cm (range)	169.5 (158–178)	167.5 (154–181)	166.5 (156–182)	166.8 (155–180)	167.5 (154–182)
Mean weight, kg (SD)	66.1 (6.3)	64.7 (7.4)	64.3 (7.2)	64.4 (6.2)	64.9 (6.8)
Median weight, kg (range)	67.8 (53.7–77.2)	65.7 (50.2–78.4)	65.3 (50.3–79.8)	64.0 (50.5–78.2)	65.4 (50.2–79.8)
Mean BMI, kg/m^2^ (SD)	23.1 (1.8)	23.1 (2.0)	22.8 (2.1)	23.0 (2.0)	23.0 (2.0)
Median BMI, kg/m^2^ (range)	23.5 (19.3–26.0)	23.1 (19.4–26.0)	22.9 (19.1–25.9)	23.3 (19.0–26.0)	23.2 (19.0–26.0)

*BMI* body mass index, *BV* bevacizumab, *CN* China, *EU* European Union, *SD* standard deviation, *US* United States

**Fig. 2:**
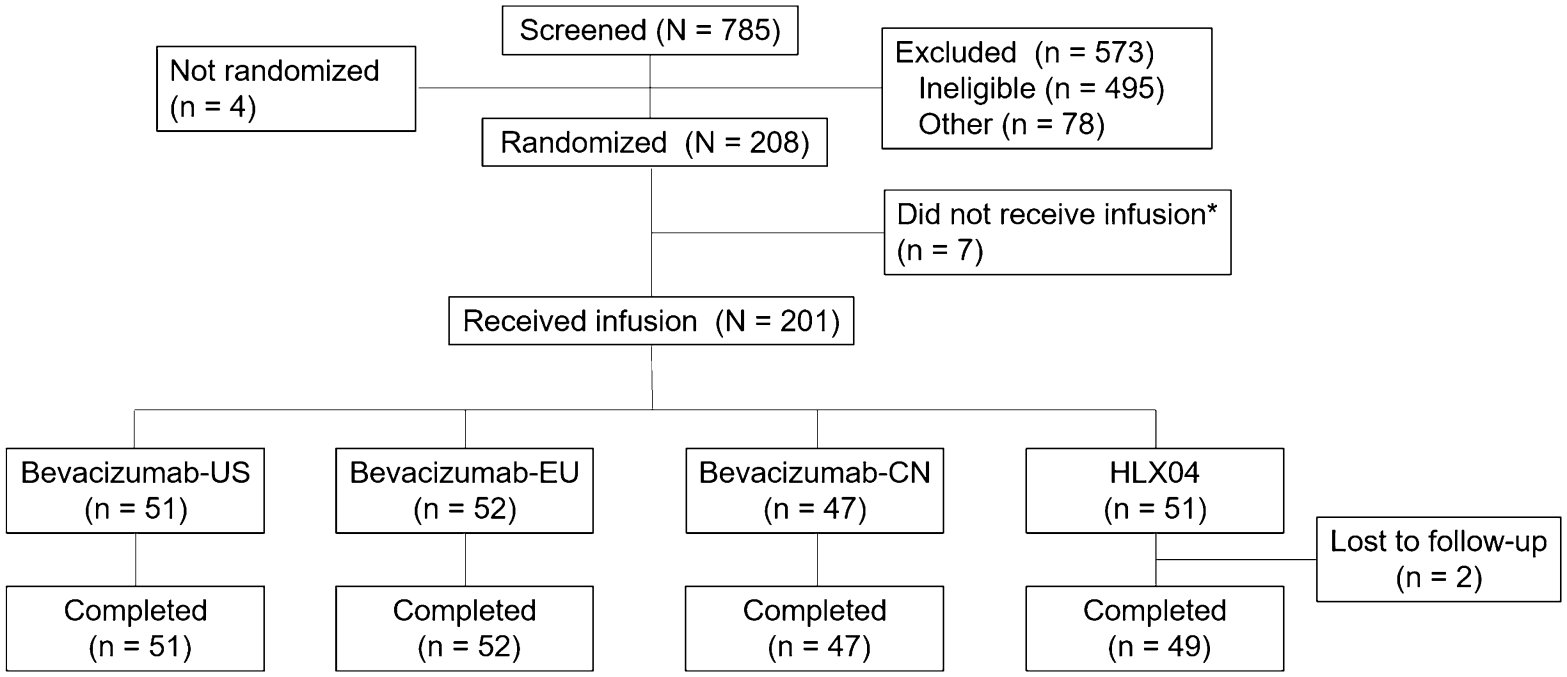
Participants disposition. *BV* bevacizumab, *CN* China, *EU* European Union, *US* United States. *Due to abdominal pain and diarrhea (HLX04, *n* = 1), or abnormal blood pressure (bevacizumab-US, *n* = 1; bevacizumab-CN, *n* = 5)

On Day 1, seven individuals did not receive treatment due to: abdominal pain and diarrhea (one participant in the HLX04 group), or abnormal blood pressure (one participant in the bevacizumab-US group and five participants in the bevacizumab-CN group). Of the 201 individuals who received treatment (safety analysis population), 199 (99%) completed the study and two individuals in the HLX04 group were lost to follow-up (Fig. 2). Thirty-one study participants reported minor protocol deviations, which were considered to have no impact on the pharmacokinetic analysis. A major protocol deviation was reported for one individual in the HLX04 group, who was lost to follow-up from Day 57 and therefore excluded from the pharmacokinetic analysis population (not excluded from the pharmacokinetic concentration population).

### Pharmacokinetics

**Fig. 3:**
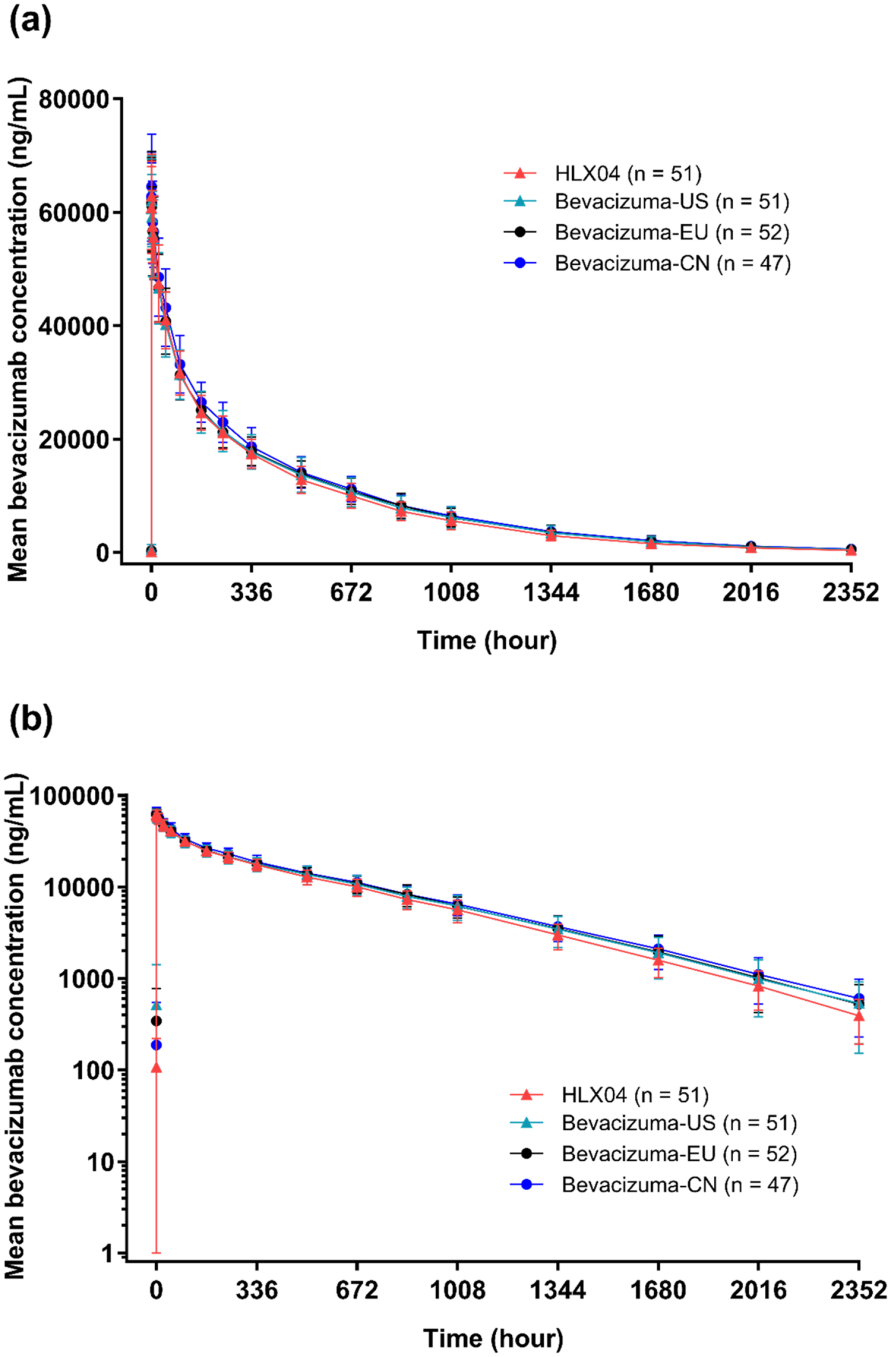
Mean (SD) serum HLX04, bevacizumab-US, bevacizumab-EU and bevacizumab-CN concentration over time: linear (**a**) and semi-logarithmic (**b**) scales (pharmacokinetic analysis population). *CN* China, *EU* European Union, *SD* standard deviation, *US* United States

Mean concentration–time profiles for each treatment group were similar over the course of the study (Fig. 3). Concentration–time profiles were characterized by a rapid decrease in serum drug concentration immediately following the end of infusion, followed by a slow elimination phase. Visual inspection of the data confirmed that the concentration–time profiles of each treatment group were superimposable.

As shown in Table 2, pharmacokinetic parameters of AUC_0–inf_, AUC_last_, C_max_, t_1/2_, t_max_, CL and V_d_ were similar in each treatment group. Table 3 showed bioequivalence assessments based on pharmacokinetic parameters for HLX04, bevacizumab-US, bevacizumab-EU and bevacizumab-CN. The two-sided 90% CIs for the test-to-reference GLSMR of AUC_0–inf_, AUC_last_ and C_max_ were within the 80–125% bioequivalence limit, confirming pharmacokinetic bioequivalence between HLX04 and reference bevacizumab from different sources (US, EU and CN).

**Table 2:** Summary of pharmacokinetic parameters (pharmacokinetic analysis population)

	HLX04 (*n* = 50)	BV-US (*n* = 51)	BV-EU (*n* = 52)	BV-CN (*n* = 47)
AUC_0–inf_, µg h/mL^a^ (CV%)	19,194 (13.9)	20,056 (20.4)	20,356 (16.6)	21,338 (16.9)
AUC_last_, µg h/mL^a^ (CV%)	18,989 (13.5)	19,778 (19.7)	20,082 (16.0)	21,005 (16.3)
C_max_, µg/mL^a^ (CV%)	64.0 (12.3)	62.9 (12.8)	63.7 (13.0)	65.2 (12.8)
Median t_max_, h (range)	2.5 (1.5–5.5)	2.5 (1.5–9.5)	2.5 (1.5–3.5)	2.5 (1.5–9.5)
Mean t_1/2,_ h (SD)	343.6 (48.0)	364.6 (69.9)	361.0 (60.3)	378.0 (65.7)
Mean CL, mL/h (SD)	10.4 (1.6)	9.8 (1.8)	9.5 (1.5)	9.1 (1.5)
Mean V_d_, L (SD)	5.2 (0.6)	5.1 (0.5)	5.1 (0.5)	4.9 (0.6)

AUC_0–inf_, area under the serum concentration curve from time 0 extrapolated to infinity

AUC_last_, area under the serum concentration curve from time 0 to the last quantifiable concentration

*BV* bevacizumab, *CL* total body clearance, *C*_*max*_ maximum observed concentration, *CN* China, *CV* coefficient of variation, *EU* European Union, *SD* standard deviation, *t*_*1/2*_ biological half-life, *t*_*max*_ time to maximum concentration, *US* United States, *V*_*d*_ volume of distribution at steady state

^a^Data are shown as geometric least-squares means

**Table 3:** Statistical comparison of key pharmacokinetic parameters (pharmacokinetic analysis population)

	GLSMR % (90% CI)		
	AUC_0–inf_	AUC_last_	C_max_
HLX04 vs BV-US	95.7 (90.4–101.3)	96.0 (90.8–101.5)	101.8 (97.7–106.1)
HLX04 vs BV-EU	94.3 (89.7–99.1)	94.6 (90.1–99.2)	100.5 (96.4–104.7)
HLX04 vs BV-CN	90.0 (85.4–94.8)	90.4 (86.0–95.1)	98.2 (94.1–102.4)
BV-US vs BV-EU	98.5 (92.8–104.7)	98.5 (92.9–104.4)	98.7 (94.6–102.9)
BV-CN vs BV-EU	104.8 (99.1–110.8)	104.6 (99.1–110.4)	102.3 (98.0–106.8)

AUC_0–inf_, area under the serum concentration curve from time 0 extrapolated to infinity

AUC_last_, area under the serum concentration curve from time 0 to the last quantifiable concentration

*BV* bevacizumab, *CI* confidence interval, *C*_*max*_ maximum observed concentration, *CN* China, *EU* European Union, *GLSMR* geometric least-squares mean ratios, *US* United States

### Safety

A total of 178 participants (88.6%) experienced at least one TEAE: 47 participants each in the HLX04 and bevacizumab-EU groups (92.2% and 90.4%, respectively), 43 (84.3%) in the bevacizumab-US group and 41 (87.2%) in the bevacizumab-CN group (Table 4). Most TEAEs were mild or moderate in intensity. Only 17 participants (8.5%) experienced a Grade 3 TEAE [4 (7.8%), 3 (5.9%), 4 (7.7%) and 6 (12.8%] participants from HLX04, bevacizumab-US, bevacizumab-EU and bevacizumab-CN groups, respectively). And one (0.5%) participant in the bevacizumab-EU group had a Grade 4 TEAE (hypertriglyceridemia).

**Table 4:** Summary of treatment-emergent adverse events (safety analysis population)

*n* (%)	HLX04 (*n* = 51)	BV-US (*n* = 51)	BV-EU (*n* = 52)	BV-CN (*n* = 47)	Total (N = 201)
Individuals with any TEAE	47 (92.2)	43 (84.3)	47 (90.4)	41 (87.2)	178 (88.6)
TEAEs by preferred term occurring in > 5% of study participants					
ALT increased	12 (23.5)	8 (15.7)	9 (17.3)	9 (19.1)	38 (18.9)
Blood CP increased	7 (13.7)	9 (17.6)	9 (17.3)	7 (14.9)	32 (15.9)
Hypertriglyceridaemia	8 (15.7)	8 (15.7)	10 (19.2)	6 (12.8)	32 (15.9)
Neutrophil count increased	11 (21.6)	7 (13.7)	9 (17.3)	1 (2.1)	28 (13.9)
AST increased	5 (9.8)	5 (9.8)	10 (19.2)	5 (10.6)	25 (12.4)
Cough	5 (9.8)	6 (11.8)	5 (9.6)	8 (17.0)	24 (11.9)
Rhinorrhoea	6 (11.8)	5 (9.8)	6 (11.5)	7 (14.9)	24 (11.9)
Leukocytosis	7 (13.7)	6 (11.8)	9 (17.3)	1 (2.1)	23 (11.4)
Oropharyngeal pain	5 (9.8)	5 (9.8)	7 (13.5)	5 (10.6)	22 (10.9)
Diarrhea	8 (15.7)	2 (3.9)	5 (9.6)	1 (2.1)	16 (8.0)
Blood bilirubin increased	3 (5.9)	5 (9.8)	2 (3.8)	4 (8.5)	14 (7.0)
Toothache	4 (7.8)	6 (11.8)	1 (1.9)	3 (6.4)	14 (7.0)
Hyperglycaemia	6 (11.8)	1 (2.0)	3 (5.8)	3 (6.4)	13 (6.5)
Headache	2 (3.9)	3 (5.9)	3 (5.8)	3 (6.4)	11 (5.5)
Hyperuricaemia	1 (2.0)	4 (7.8)	3 (5.8)	3 (6.4)	11 (5.5)
Rash	4 (7.8)	3 (5.9)	1 (1.9)	2 (4.3)	10 (5.0)
Gingival swelling	3 (5.9)	1 (2.0)	2 (3.8)	3 (6.4)	9 (4.5)
Sinus bradycardia	3 (5.9)	0 (0.0)	4 (7.7)	1 (2.1)	8 (4.0)
WBC urine positive	1 (2.0)	2 (3.9)	3 (5.8)	2 (4.3)	8 (4.0)
Abdominal pain	3 (5.9)	0 (0.0)	2 (3.8)	2 (4.3)	7 (3.5)
Abdominal pain upper	2 (3.9)	1 (2.0)	0 (0.0)	4 (8.5)	7 (3.5)
Hypertension	2 (3.9)	1 (2.0)	3 (5.8)	1 (2.1)	7 (3.5)
WBC count increased	4 (7.8)	1 (2.0)	1 (1.9)	0 (0.0)	6 (3.0)
Nausea	0 (0.0)	0 (0.0)	3 (5.8)	1 (2.1)	4 (2.0)
Mild (Grade 1)	30 (58.8)	27 (52.9)	28 (53.8)	27 (57.4)	112 (55.7)
Moderate (Grade 2)	13 (25.5)	13 (25.5)	14 (26.9)	8 (17.0)	48 (23.9)
Severe (Grade 3)	4 (7.8)	3 (5.9)	4 (7.7)	6 (12.8)	17 (8.5)
Life threatening (Grade 4)	0 (0.0)	0 (0.0)	1 (1.9)	0 (0.0)	1 (0.5)
Death (Grade 5)	0 (0.0)	0 (0.0)	0 (0.0)	0 (0.0)	0 (0.0)
Individuals with any serious TEAE	0 (0.0)	1 (2.0)	0 (0.0)	3 (6.4)	4 (2.0)
Individuals with ADRs	42 (82.4)	38 (74.5)	39 (75.0)	34 (72.3)	153 (76.1)

*ADRs* adverse drug reactions, *ALT* alanine aminotransferase, *AST* aspartate aminotransferase, *BV* bevacizumab, *CN* China, *CP* creatine phosphokinase, *EU* European Union, *TEAE* treatment-emergent adverse event, *US* United States, *WBC* white blood cell

The most common TEAEs by preferred term were alanine aminotransferase increased, blood creatine phosphokinase increased and hypertriglyceridaemia. All incidence of alanine aminotransferase increased and neutrophil count increased were Grade 1. Grade 3 blood creatine phosphokinase increased was reported in four individuals: one in both bevacizumab-US and bevacizumab-CN groups and two in bevacizumab-EU group (Supplementary table 2). Serious TEAEs occurred in one individual in the bevacizumab-US group (Grade 3 cataract) and in three individuals in the bevacizumab-CN group (Grade 3 appendicitis, Grade 3 ophthalmic herpes simplex and Grade 3 tuberculous pleurisy). There were no Grade 5 TEAEs or deaths during the study. Mild infusion-site reactions occurred in three individuals (one each in the HLX04, bevacizumab-CN and bevacizumab-EU groups). The incidence of adverse drug reaction (ADR) was generally comparable among treatment groups (Table 4). Full ADRs in treatment groups were listed in Supplementary table 3.

No clinically meaningful mean changes from baseline were observed for any laboratory parameter in any treatment group, except for triglycerides. In the HLX04 group, 0.531 mmol/L mean increase from baseline was detected on Day 3, which was similar to those of the other three reference groups. On Day 1, following intravenous infusion of study products, decreases from baseline were observed in all four treatment groups for systolic blood pressure, diastolic blood pressure, pulse rate and temperature. Decreases were of similar magnitude across groups and mean values returned to baseline levels on Day 2 and remained stable until the end of the study. Most participants (> 90%) had normal or clinically insignificant changes in 12-lead ECG at each visit, with no notable differences observed among the four treatment groups.

### Immunogenicity

ADAs were not detected in any of the individual samples at any time point during the study.

## Discussion

The availability of biosimilar increases the treatment options available to physicians and facilitates patient access to effective therapies. HLX04 was developed as a bevacizumab biosimilar in the same indications as the reference product, and was produced by recombinant DNA technology using the same expression system (Chinese hamster ovary cells) as reference bevacizumab. In a rigorous preclinical evaluation, HLX04 was shown to be comparable to bevacizumab in terms of its physicochemical and functional properties [28]. Pharmacokinetic (PK) studies in cynomolgus monkeys showed that HLX04 had very similar PK characteristics and identical toxicokinetic characteristics as reference bevacizumab at doses of 2–50 mg/kg [28]. This randomized, double-blind, four-arm, parallel-controlled, Phase 1 study was designed to establish the bioequivalence of proposed bevacizumab biosimilar HLX04 to reference bevacizumab in healthy Chinese males, with respect to pharmacokinetic parameters, safety and immunogenicity.

Here, we demonstrate bioequivalence between HLX04 and reference bevacizumab, as the GLSMR of AUC_0–inf_, AUC_last_ and C_max_ fulfilled the standard equivalence criteria [33]. The treatment ratios for all three measures of systemic exposure were near to one unity (90.0‒104.8%), and the lower and upper 90% CIs were within 15% of unity for all pairwise comparisons. There was no apparent difference in t_max_, t_1/2_, CL or V_d_ across the four products. The reference bevacizumab products from different sources (US, EU and CN) were shown to be bioequivalent to each other in terms of pharmacokinetics and safety.

The 3 mg/kg dose used in the present study was within the linear pharmacokinetic range established for bevacizumab (allowing results to be extrapolated to the standard clinical dose) [34], and was the same dose used in other Phase 1 healthy volunteer studies of bevacizumab biosimilar candidates [13, 15, 16, 18, 21]. In line with previous Phase 1 bevacizumab biosimilar trials, healthy males were chosen as a suitable homogenous population to detect differences in pharmacokinetic profiles, to avoid potential confounding effects caused by disease or concomitant medication that may interfere with results. The GLSMR demonstrated in this study were in line with those seen in other Phase 1 bevacizumab pharmacokinetic bioequivalence studies [15].

No notable differences in the frequency, nature and/or grade of TEAEs were observed among the four treatment groups in this study. No deaths occurred during the study. Furthermore, no ADAs were detected in any individuals. These safety findings were consistent with those reported for bevacizumab biosimilars administered at the same dose [13, 15, 16, 18, 21]. One limitation was that only Chinese male subjects were included in this study to minimize the effects of physiological differences, which affect the PK evaluations between the study drug and the reference product. Another limitation was the relatively short study duration. Thus, further safety investigation of HLX04 among larger and diverse populations with longer duration is necessary.

A major strength of this study was that three reference products from different sources (US, EU, and CN) were used, allowing five different pairwise comparisons to be carried out. This ensured that pharmacokinetic bioequivalence could be demonstrated thoroughly between HLX04 and the three reference products, as well as between one reference product and another. The result also supported further clinical evaluations of HLX04 to a reference product in a phase 3 study.

In conclusion, pharmacokinetic bioequivalence of HLX04 to reference bevacizumab-US, bevacizumab-EU and bevacizumab-CN in healthy Chinese males was demonstrated. No clinically meaningful differences were observed between HLX04 and reference bevacizumab products in safety and immunogenicity. The comparative efficacy and safety of HLX04 and reference bevacizumab, combined with oxaliplatin and fluoropyrimidine-based chemotherapy (XELOX or mFOLFOX6), are being assessed in a randomized, double-blind, multi-center Phase 3 study involving approximately 640 Chinese patients with previously untreated metastatic colorectal cancer (NCT03511963).

## Supplementary Information

Below is the link to the electronic supplementary material.

Supplementary file1 (DOCX 27 KB)

## Data Availability

The data that support the findings of this study are available from the corresponding author upon reasonable request.
