# Supplementary material for: A phase 1 randomized study compare the pharmacokinetics, safety and immunogenicity of HLX04 to reference bevacizumab sourced from the United States, the European Union, and China in healthy Chinese male volunteers
Source: Cancer Chemother Pharmacol. 2021 Jun 4;88(3):465–74. doi: 10.1007/s00280-021-04297-z (PMC8316231; doi:10.1007/s00280-021-04297-z)
Supplement: Supplementary file 1 — Supplementary file1 (DOCX 27 KB) [file 280_2021_4297_MOESM1_ESM.docx]

**Supplementary materials**

**Supplementary table 1** Inclusion and Exclusion Criteria:

| Inclusion Criteria: |
| --- |
| 1. Subjects who were willing to comply with the contraception restrictions for this study 2. Subjects who were able and willing to give written informed consent 3. Subjects who were willing to comply with the study restrictions from screening until end of study 4. Adult males aged 18 to 50 years inclusive and between 19 and 26 kg/m^2^ body mass index (BMI) and body weight ≥50 kg and ≤80 kg 5. Subjects who were non-smokers or had not used tobacco or nicotine containing products for at least 3 months prior to screening and had less than 5 cigarettes per day smoking history. Subjects agreed to refrain from smoking during days of confinement at the study center 6. Subjects negative for Hepatitis B surface antigen, Hepatitis C virus antibody, treponema pallidum antibody, and human immunodeficiency virus antibody tests 7. Subjects negative for urine drug screen and alcohol tests 8. Subjects determined healthy by medical history, physical examination, laboratory tests, 12-lead electrocardiogram (ECG), and chest X-ray, without any clinically significant abnormality judged by the Investigator |
| Exclusion Criteria: |
| 1. History of gastrointestinal, endocrine, pulmonary, hepatic, renal, psychiatric, neurological, cardiovascular, hematological, and metabolic (including known diabetes mellitus) disease or disorder considered as significant by the Investigator 2. History of any cancer, lymphoma, or leukemia, except basal cell carcinoma of skin after localized cancer was removed 3. History or current clinically significant atopic allergy, hypersensitivity or allergic reactions including known or suspected clinically relevant drug hypersensitivity to any component of the investigational product or reference product formulations 4. Any disorder that, in the Investigator’s opinion, interfered with the safety of the subject and the study procedures and evaluations 5. Blood loss or blood donation (including blood components donation) ≥400 mL or blood transfusion within 3 months before screening; blood loss or blood donation (including blood components donation) ≥200 mL within 1 months prior to screening 6. Surgery within the past 8 weeks or surgery planned during the study duration 7. Poor oral hygiene that required surgical intervention during the study or any planned dental interventions during the study 8. Live virus vaccination within 4 weeks prior to screening or intention to receive live virus vaccination during the study until the final follow-up visit 9. History of prior exposure to bevacizumab or any anti-vascular endothelial growth factor (VEGF) or anti-VEGF receptor (VEGFR) monoclonal antibodies or proteins (e.g. aflibercept, ramucirumab, lapatinib, and sunitinib) 10. Prior exposure to any other investigational monoclonal antibody within 12 months of the single dose administration 11. Use of any investigational drug in any clinical study within the 3 months prior to the single dose administration in this study; or remained on follow-up of any clinical study 12. Any intake of a non-steroidal anti-inflammatory drug (NSAID) including any dose of aspirin in the last 14 days. Non-steroidal anti-inflammatory drugs were not allowed for the duration of the study. Paracetamol was allowed for pain control if required 13. Intake of prescribed or over-the-counter drugs within 28 days of the single dose administration or herbal drugs or dietary supplements within 28 days prior to the single dose administration 14. Any persons who were:     1. An employee of the Principle Investigators, study centers, contract research organization (CRO), or the Sponsor     2. A relative of an employee of the study centers, the Investigators, CRO, or the Sponsor 15. Abnormal ECG with clinical significance judged by the Investigator 16. Abnormal serum Immunoglobulin G with clinical significance judged by the Investigator 17. Confirmed positive ADA at screening 18. Occurrence of acute disease during screening or predose, e.g. acute hepatitis, acute diarrhea 19. Intake of any product containing alcohol within 24 hours of the single dose administration 20. Subjects with relevant family history of hypertension or abnormal blood pressure at screening or admission to the study center (Day 1) 21. Systolic blood pressure >40 mmHg 22. Diastolic blood pressure >90 mmHg 23. Any inherited predisposition to bleeding or to thrombosis or history of non-traumatic hemorrhage (ie, requiring medical intervention), thromboembolic event or any condition which increased bleeding risk including clotting disorders, thrombocytopenia (platelet count <100000/μL) or an international normalized ratio higher than 1.5 24. Any clinically significant infection ongoing at screening or admission to the Phase I clinical trial unit 25. Total cholesterol >1.5 × upper limit of normal or fasting glucose abnormality with clinical significance at screening or admission 26. History of alcohol abuse or a positive alcohol breath test, history of drug abuse or positive urine drug screen |

**Supplementary table 2** Graded treatment-emergent blood creatine phosphokinase

| **Preferred term**  **Severity Grade** | **HLX04**  **(n = 51)**  **n (%)** | **BV-US**  **(n = 51)**  **n (%)** | **BV-EU**  **(n = 52)**  **n (%)** | **BV-CN**  **(n = 47)**  **n (%)** | **Total**  **(n = 201)**  **n (%)** |
| --- | --- | --- | --- | --- | --- |
| Blood creatine phosphokinase increased | 7 (13.7) | 9 (17.6) | 9 (17.3) | 7 (14.9) | 32 (15.9) |
| Grade 1 | 6 (11.8) | 7 (13.7) | 2 (3.8) | 5 (10.6) | 20 (10.0) |
| Grade 2 | 1 (2.0) | 1 (2.0) | 5 (9.6) | 1 (2.1) | 8 (4.0) |
| Grade 3 | 0 (0.0) | 1 (2.0) | 2 (3.8) | 1 (2.1) | 4 (2.0) |
| Grade 4 | 0 (0.0) | 0 (0.0) | 0 (0.0) | 0 (0.0) | 0 (0.0) |
| Grade 5 | 0 (0.0) | 0 (0.0) | 0 (0.0) | 0 (0.0) | 0 (0.0) |

BV, bevacizumab; US, United States; EU, European Union; CN, China.

**Supplementary table 3** Adverse drug reactions with incidence rates ≥5% of all subjects (safety analysis population)

| **System organ class**  **Preferred term** | **HLX04**  **(n = 51)**  **n (%)** | **BV-US**  **(n = 51)**  **n (%)** | **BV-EU**  **(n = 52)**  **n (%)** | **BV-CN**  **(n = 47)**  **n (%)** | **Total**  **(N = 201)**  **N(%)** |
| --- | --- | --- | --- | --- | --- |
| Number of Subjects with at least 1 ADR | 42 (82.4) | 38 (74.5) | 39 (75.0) | 34 (72.3) | 153 (76.1) |
| Total number of ADRs | 129 | 105 | 131 | 88 | 453 |
| Investigations |  |  |  |  |  |
| Number of Subjects | 23 (45.1) | 25 (49.0) | 29 (55.8) | 20 (42.6) | 97 (48.3) |
| Total number of ADRs | 54 | 52 | 62 | 35 | 203 |
| Alanine aminotransferase increased | 12 (23.5) | 8 (15.7) | 9 (17.3) | 9 (19.1) | 38 (18.9) |
| Aspartate aminotransferase increased | 5 (9.8) | 5 (9.8) | 10 (19.2) | 5 (10.6) | 25 (12.4) |
| Blood creatine phosphokinase increased | 6 (11.8) | 7 (13.7) | 5 (9.6) | 6 (12.8) | 24 (11.9) |
| Neutrophil count increased | 8 (15.7) | 6 (11.8) | 8 (15.4) | 0 (0.0) | 22 (10.9) |
| Blood bilirubin increased | 3 (5.9) | 4 (7.8) | 1 (1.9) | 3 (6.4) | 11 (5.5) |
| Gastrointestinal disorders  Number of Subjects | 16 (31.4) | 7 (13.7) | 12 (23.1) | 11 (23.4) | 46 (22.9) |
| Total number of ADRs | 22 | 8 | 16 | 17 | 63 |
| Diarrhoea | 8 (15.7) | 1 (2.0) | 5 (9.6) | 1 (2.1) | 15 (7.5) |
| Metabolism and nutrition disorders  Number of Subjects | 12 (23.5) | 12 (23.5) | 12 (23.1) | 8 (17.0) | 44 (21.9) |
| Total number of ADRs | 19 | 21 | 22 | 13 | 75 |
| Hypertriglyceridaemia | 4 (7.8) | 6 (11.8) | 8 (15.4) | 5 (10.6) | 23 (11.4) |
| Hyperuricaemia | 1 (2.0) | 4 (7.8) | 3 (5.8) | 3 (6.4) | 11 (5.5) |
| Blood and lymphatic system disorders  Number of Subjects | 6 (11.8) | 5 (9.8) | 8 (15.4) | 0 (0.0) | 19 (9.5) |
| Total number of ADRs | 9 | 5 | 11 | 0 | 25 |
| Leukocytosis | 6 (11.8) | 5 (9.8) | 8 (15.4) | 0 (0.0) | 19 (9.5) |
| Nervous system disorders  Number of Subjects | 4 (7.8) | 4 (7.8) | 3 (5.8) | 4 (8.5) | 15 (7.5) |
| Total number of ADRs | 4 | 4 | 3 | 4 | 15 |
| Headache | 2 (3.9) | 3 (5.9) | 2 (3.8) | 3 (6.4) | 10 (5.0) |
| Skin and subcutaneous tissue disorders  Number of Subjects | 6 (11.8) | 4 (7.8) | 3 (5.8) | 2 (4.3) | 15 (7.5) |
| Total number of ADRs | 7 | 5 | 3 | 2 | 17 |
| Rash | 4 (7.8) | 3 (5.9) | 1 (1.9) | 2 (4.3) | 10 (5.0) |

BV, bevacizumab; US, United States; EU, European Union; CN, China; ADRs, adverse drug reactions.
